# Supplementary material for: Spinal cord stimulation modulates post-synaptic inhibition improving neuromotor control of arm movement in people with chronic hemiparesis post-stroke
Source: medRxiv. 2025 Sep 29:2025.09.23.25336157. Preprint. [Version 1] doi: 10.1101/2025.09.23.25336157 (PMC12622153; doi:10.1101/2025.09.23.25336157)
Supplement: Supplement 1 [file NIHPP2025.09.23.25336157v1-supplement-1.pdf]

medRxiv preprint doi: https://doi.org/10.1101/2025.09.23.25336157; this version posted September 29, 2025. The copyright holder has placed this preprint (which was not certified by peer review) in the Public Domain. It is no longer restricted by copyright. Anyone can legally share, reuse, remix, or adapt this material for any purpose without crediting the original authors.

Supplemental information

Extended data figure 1

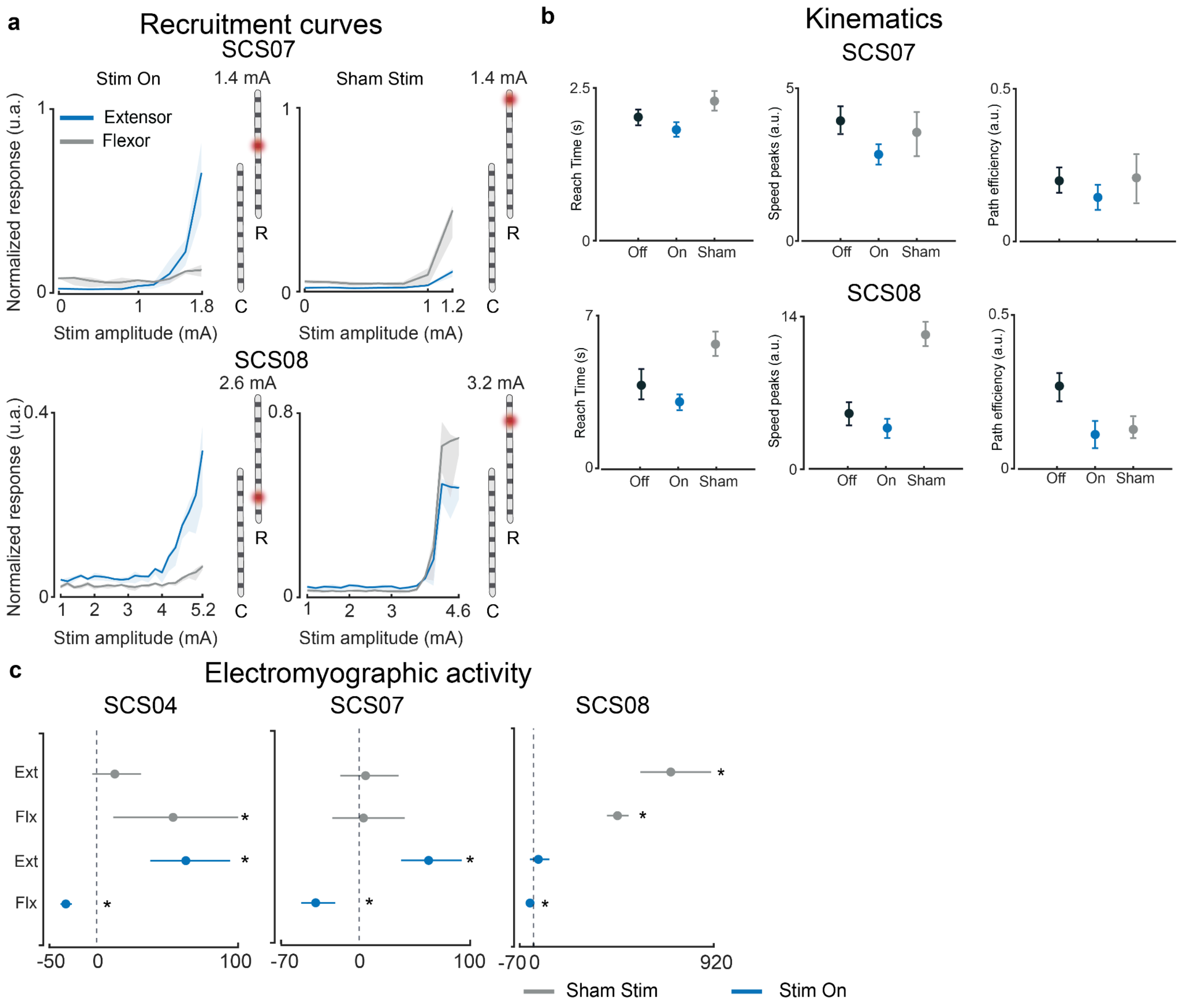

medRxiv preprint doi: https://doi.org/10.1101/2025.09.23.25336157; this version posted September 29, 2025. The copyright holder has placed this preprint (which was not certified by peer review) in the Public Domain. It is no longer restricted by copyright. Anyone can legally share, reuse, remix, or adapt this material for any purpose without crediting the original authors.

Extended data figure 1 | Planar reaching kinematics and muscle activity: a, Top: recruitment curves for contact 4R (left) and contact 1R (right) and normalized evoked responses for triceps brachii (blue, elbow extensor) and biceps brachii (gray, elbow flexor). Bottom: recruitment curves for contact 7R (left) and contact 2R (right) and normalized evoked responses for triceps brachii (blue, elbow extensor) and biceps brachii (gray, elbow flexor). **b**,Quantification of the mean values of the kinematics metrics used to assess the effect of SCS (On) and Sham stimulation with respect to stimulation Off for SCS07 (top) and SCS08 (bottom). **c**, Quantification of percent change of the muscles EMG activity with respect to stimulation Off (EXT: triceps brachii with stimulation On (blue) and Sham (gray) stimulation; FLX: brachioradialis (SCS04, SCS07), biceps brachii (SCS08) with agonist (blue) and Sham (gray) stimulation). The middle circle on plots indicates the mean value of the measurements. All error bars indicate the 95% confidence interval computed with bootstrap N=10,000. The single asterisk indicates statistical significance and rejection of the null hypothesis of no difference with a 95% CI.

medRxiv preprint doi: https://doi.org/10.1101/2025.09.23.25336157; this version posted September 29, 2025. The copyright holder has placed this preprint (which was not certified by peer review) in the Public Domain. It is no longer restricted by copyright. Anyone can legally share, reuse, remix, or adapt this material for any purpose without crediting the original authors.

Extended data figure 2

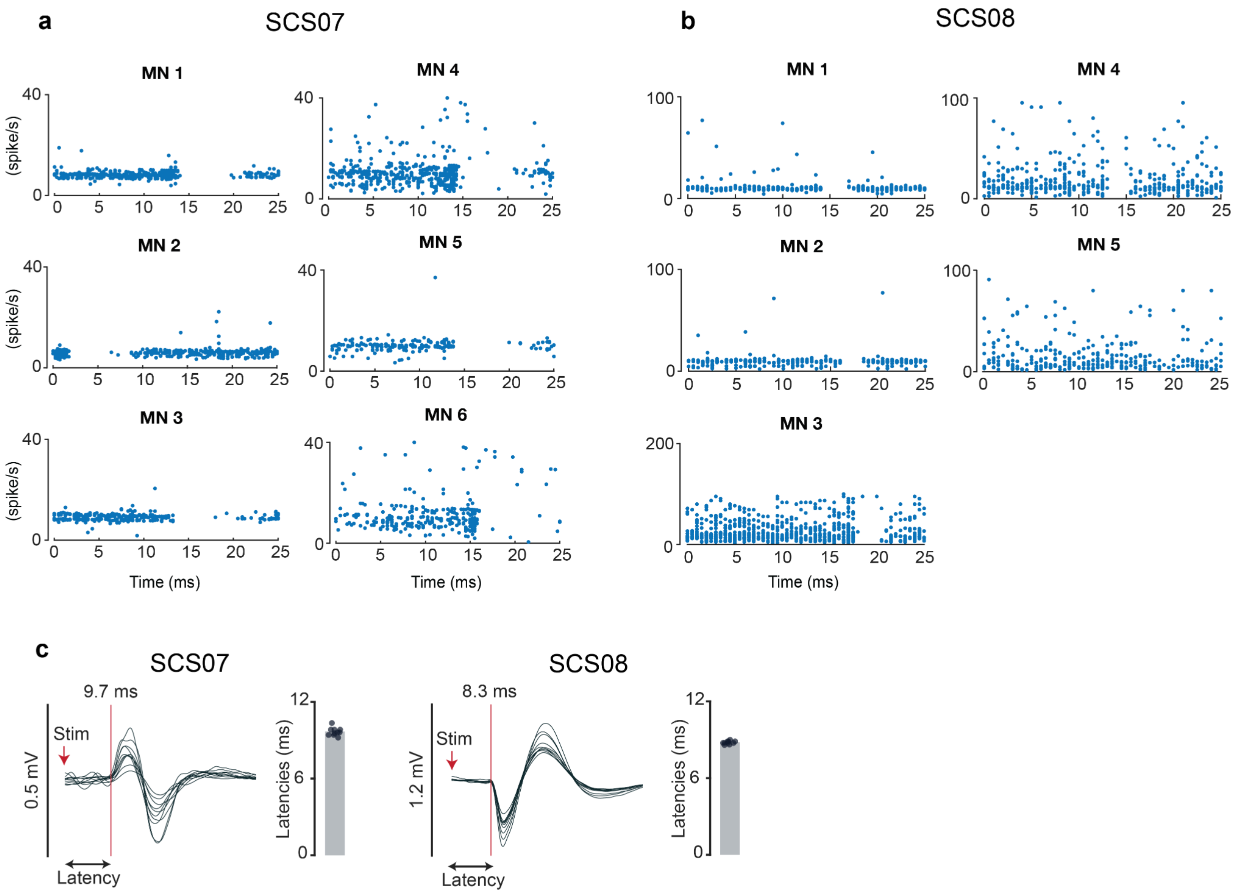

Extended data figure 2 | Peristimulus frequencygram (PSF) and action potential latencies: a,**b** Peristimulus frequencygram of all the detected motoneurons (MNs) for SCS07 and SCS08, respectively. Circles on plots indicate the instantaneous firing rate of the MNs at the reported delay from the SCS pulses. The interval considered spans 0 ms to 25 ms post-stimulus, which is the stimulation time period for the 40 Hz tonic SCS used in this experiment. **c**, Latencies of the PRM-reflexes evoked in the biceps brachii as an estimate of the action potential propagation delay for SCS07 and SCS08. Bar plots show the mean value of the action potential delay with respect to the SCS pulses. Circles on plots indicate the time delay between the stimulation pulse and the recorded action potential for n = 10 evoked responses. Evoked responses were recorded at rest while delivering supra-threshold SCS targeting Ia sensory afferents innervating Biceps Brachii.
